# Thinking outside the ‘health’ box: interventions for malaria control

**DOI:** 10.1186/1475-2875-11-S1-O41

**Published:** 2012-10-15

**Authors:** Steve Lindsay

**Affiliations:** 1 Durham University, Science Laboratories, South Road, Durham DH1 3LE, UK

## 

In the last decade malaria has declined sharply in many countries in the tropics, due largely to the massive expansion of control programmes. However, the future may not be as bright as drug and insecticide resistance rises and aid budgets constrict. Since it is well recognised that malaria declined in many parts of the world due to socio-economic improvements, here I consider whether development today could be an effective weapon against malaria.

